# Retroperitoneal Soft Tissue Sarcoma: Emerging Therapeutic Strategies

**DOI:** 10.3390/cancers15225469

**Published:** 2023-11-18

**Authors:** Eelco de Bree, Dimosthenis Michelakis, Ioannis Heretis, Nikolaos Kontopodis, Konstantinos Spanakis, Eleni Lagoudaki, Maria Tolia, Michail Zografakis-Sfakianakis, Christos Ioannou, Dimitrios Mavroudis

**Affiliations:** 1 Department of Surgical Oncology, Medical School of Crete University Hospital, 71110 Heraklion, Greece; dimosthenis@msn.com; 2 Department of Urology, Medical School of Crete University Hospital, 71110 Heraklion, Greece; gheretis@yahoo.gr; 3 Department of Vascular Surgery, Medical School of Crete University Hospital, 71110 Heraklion, Greece; nkontopodis@uoc.gr (N.K.); ioannou@uoc.gr (C.I.); 4 Department of Medical Imaging, Medical School of Crete University Hospital, 71110 Heraklion, Greece; vispan@windowslive.com; 5 Department of Pathology, Medical School of Crete University Hospital, 71110 Heraklion, Greece; elagoudakimd@gmail.com; 6 Department of Radiation Oncology, Medical School of Crete University Hospital, 71110 Heraklion, Greece; mariatolia@uoc.gr; 7 Department of Nursing, School of Health Sciences, Hellenic Mediterranean University, 71110 Heraklion, Greece; mzografakis@hmu.gr; 8 Department of Medical Oncology, Medical School of Crete University Hospital, 71110 Heraklion, Greece; mavroudis@uoc.gr

**Keywords:** retroperitoneal sarcoma, multimodality management, surgery, radiotherapy, chemotherapy

## Abstract

**Simple Summary:**

Malignant soft tissue tumours rarely arise in the space between the peritoneum and the posterior abdominal wall that contains many significant organs and structures. These retroperitoneal soft tissue sarcomas (RPSs) are mainly treated with surgery, but since wide resection is usually unfeasible, they frequently recur due to its large size, indistinct tumour borders, anatomic constraints and the thinness of the overlying peritoneum. In recent decades, the role of emerging therapeutic strategies, such as more extended surgery and administration of radiotherapy and/or chemotherapy before or after surgery, to improve oncological outcome in primary localised RPS has been extensively investigated. In this review, the recent data on the evolving multidisciplinary management of primary localised RPS are comprehensively discussed. The heterogeneity of RPS, with their different histological subtypes and biological behaviour, renders a standard therapeutic ‘one-size-fits-all’ approach inappropriate, and treatment should be modified according to histological type and malignancy grade of these tumours.

**Abstract:**

Retroperitoneal soft tissue sarcoma (RPS) is a rare and heterogenous disease for which surgery is the cornerstone of treatment. However, the local recurrence rate is much higher than in soft tissue sarcoma of the extremities since wide resection is usually unfeasible in RPS due to its large size, indistinct tumour borders, anatomical constraints and the thinness of the overlying peritoneum. Local recurrence is the leading cause of death for low-grade RPS, whereas high-grade tumours are prone to distant metastases. In recent decades, the role of emerging therapeutic strategies, such as more extended surgery and (neo)adjuvant treatments to improve oncological outcome in primary localised RPS, has been extensively investigated. In this review, the recent data on the evolving multidisciplinary management of primary localised RPS are comprehensively discussed. The heterogeneity of RPS, with their different histological subtypes and biological behaviour, renders a standard therapeutic ‘one-size-fits-all’ approach inappropriate, and treatment should be modified according to histological type and malignancy grade. There is sufficient evidence that frontline extended surgery with compartmental resection including all ipsilateral retroperitoneal fat and liberal en bloc resection of adjacent organs and structures, even if they are not macroscopically involved, increases local tumour control in low-grade sarcoma and liposarcoma, but not in leiomyosarcoma for which complete macroscopic resection seems sufficient. Additionally, preoperative radiotherapy is not indicated for all RPSs, but seems to be beneficial in well-differentiated liposarcoma and grade I/II dedifferentiated liposarcoma, and probably in solitary fibrous tumour. Whether neoadjuvant chemotherapy is of benefit in high-grade RPS remains unclear from retrospective data and is subject of the ongoing randomised STRASS 2 trial, from which the results are eagerly awaited. Personalised, histology-tailored multimodality treatment is promising and will likely further evolve as our understanding of the molecular and genetic characteristics within RPS improves.

## 1. Introduction

Sarcomas are relatively rare tumours that may be localised at any site of the human body. Approximately 10–15% of all soft tissue sarcomas (STSs) are found in the retroperitoneum. The estimated incidence of retroperitoneal soft tissue sarcomas (RPSs) is approximately 0.3–0.5 new cases per 100,000 inhabitants per year [1,2,3]. Their localisation, more expansive than infiltrative manner of growth, slow growth and low metastatic potential usually result in late symptoms presentation. Therefore, they usually reach a large size when belatedly diagnosed. However, RPS is not a single disease and encompasses a heterogenous group of tumours with different biological behaviour, response to treatment and oncological risk. The most common histological subtypes encountered, according to a combined analysis of two large RPS databases, are well-differentiated liposarcoma (24%), dedifferentiated liposarcoma (40%), leiomyosarcoma (20%), solitary fibrous tumour (5%) and malignant peripheral nerve sheath tumour (3%) [4].

Surgery is the mainstay of curative treatment, with local control (LC) being essential for the patient’s outcome, especially in low-grade sarcomas that have a minimal metastatic potential [4,5]. Generally, while the aim in sarcoma surgery is the achievement of wide surgical margins, in RPS this is hindered by the large size of the tumour, the anatomical constraints in the retroperitoneal space, indistinct borders and thinness of the peritoneum covering these tumours. Consequently, local recurrence (LR) is traditionally more frequently observed after surgery for primary RPS than for extremity STS [6,7,8,9,10,11,12,13,14,15,16,17,18,19]. Unlike the case for extremity STSs and most malignancies, LR comprises the leading cause of death for RPSs since most are low- to intermediate-grade liposarcomas with very low metastatic biological behaviour [5,7,20]. In well-differentiated and dedifferentiated liposarcoma, the 10-year LR rates are 51% and 62%, respectively, and the 5-year distant recurrence rates are 8% and 28%, respectively [18,19]. The feasibility of surgery and the local disease-free interval decreases with each further LR, and the patients eventually develop extensive abdominal disease which is incurable even with contemporary multidisciplinary approaches. Leiomyosarcoma, a frequent and high-grade histological subtype, is prone to distant metastases (DM) (5-year distant recurrence 50%) and the risk of LR after complete resection is of less importance (10-year LR 25%) [5,17,18,19]. Therefore, it is clear that the pattern of recurrence and survival are histology specific [17,18,19,20,21,22].

During the last one and a half decade, there has been strong effort to define therapeutic strategies to reduce the high overall 5-year LR rate of approximately 40–60% and to improve the poor overall 5-year overall survival (OS) of approximately 40–65% after traditional treatment of primary RPS [6,7,8,9,10,11,12]. The heterogeneity of RPS, with differences in biological behaviour and sensitivity to (neo)adjuvant treatments, renders a standard therapy for all RPSs inappropriate and suggests a histology-tailored multidisciplinary approach. Due to the rarity and heterogeneity of RPS, the number of randomised trials is very limited and additional evolving evidence for optimal treatment of primary RPS is provided by retrospective studies, whereas international expert consensuses may provide clinical practice guidance [4,23,24]. In this comprehensive review, the emerging therapeutic strategies to improve the outcome of primary localised RPS are thoroughly discussed.

## 2. Diagnosis and Pre-Treatment Evaluation

**Figure 1:**
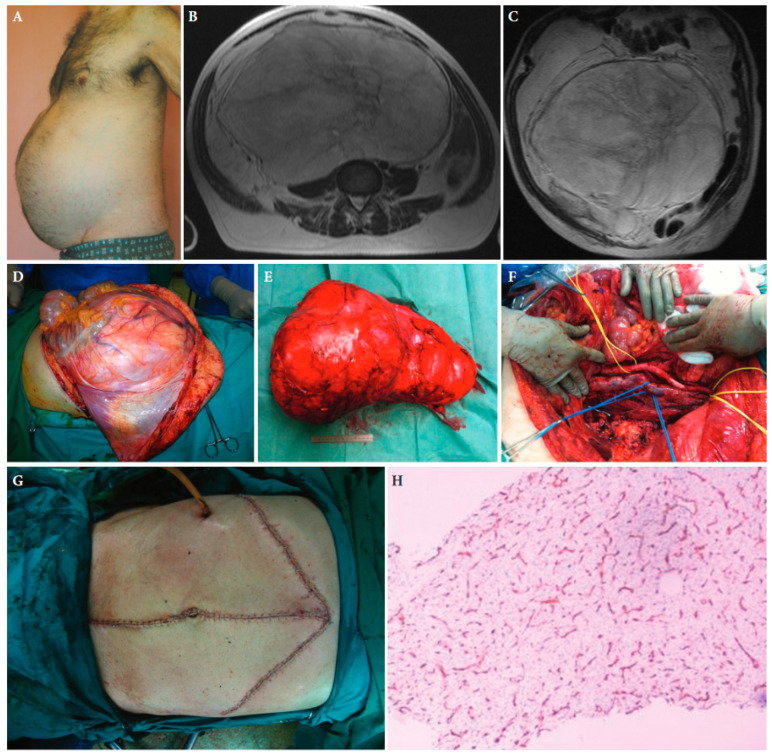
A 55-year-old male patient with retroperitoneal sarcoma as the only symptom, weighting 13 kg and measuring 45 cm in maximal diameter, and abdominal distension (**A**). (**B**,**C**): Magnetic resonance imaging showing the tumour. (**D**): The large tumour at laparotomy. (**E**): The surgical specimen. (**F**): The abdominal cavity after resection of the tumour. (**G**): The medial laparotomy with bilateral subcostal incisions required to resect the tumour completely. (**H**): Histopathologic examination of the tumour established the diagnosis of a low-grade myxoid liposarcoma. CD34 immunohistochemical staining of a tumour tissue slide highlighting the characteristic delicate thin-walled arborizing and curving capillaries that form a network reminiscent of chicken wirefencing, ×10 magnification ([25] with permission).

**Figure 2:**
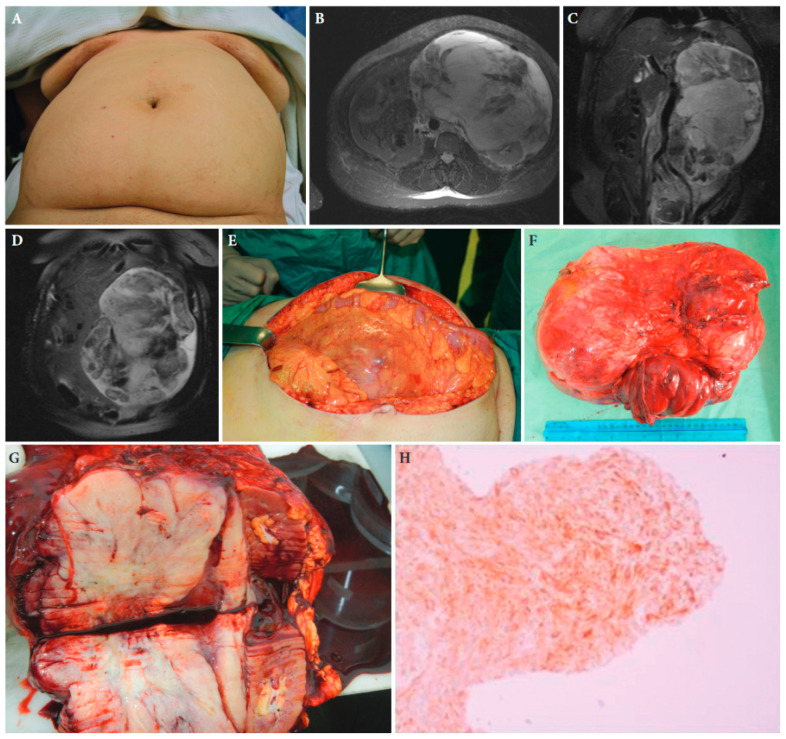
A 52-year-old female patient with retroperitoneal sarcoma as the only symptom, measuring 29 cm in maximal diameter, and abdominal distension (**A**). (**B**–**D**): Magnetic resonance imaging showing the tumour. (**E**): The large tumour at laparotomy. (**F**,**G**): The surgical specimen of the tumour with adjacent kidney and adrenal gland. (**H**): Histopathologic examination of the tumour established the diagnosis of an extra-gastrointestinal stromal tumour. Tissue slide of the tumour demonstrating CD34 immunohistochemical expression, ×40 magnification ([25] with permission).

As previously mentioned, RPS generally grows slowly and reaches a large size before being diagnosed. RPS is asymptomatic for a prolonged period and is sometimes revealed incidentally during imaging for other reasons. When symptomatic, the symptoms and signs are mostly nonspecific and include abdominal discomfort or pain, and abdominal distension or mass (Figure 1 and Figure 2). Less frequently, these tumours cause symptoms associated with obstruction or infiltration of the gastrointestinal or urinary tract.

**Figure 3:**
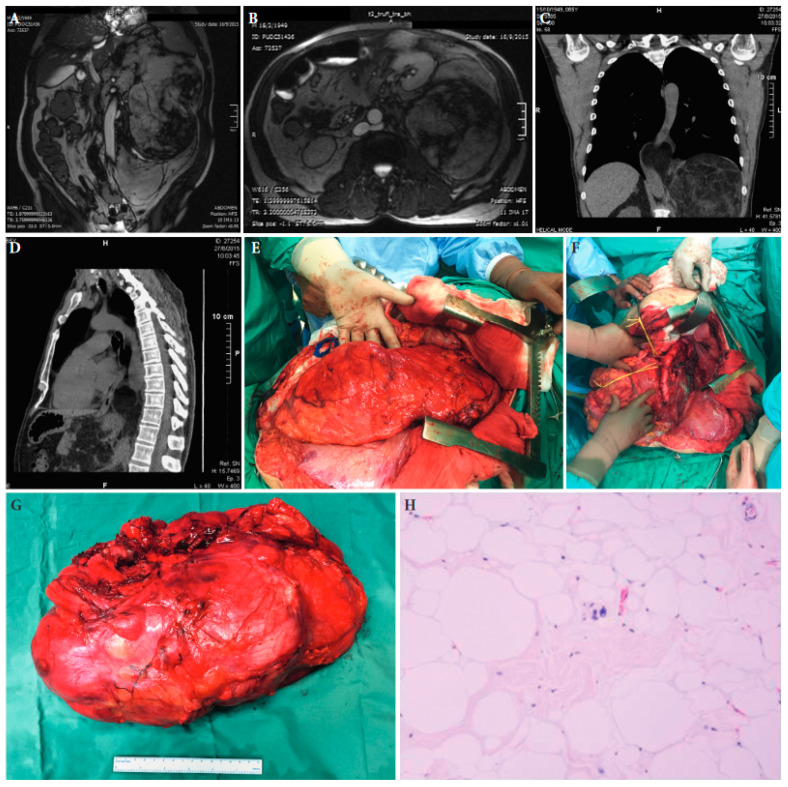
A 67-year-old male patient with a large retroperitoneal sarcoma and displacement of the left kidney just under the abdominal wall at magnetic resonance imaging (**A**,**B**) and extension into the chest at chest computed tomography (**C**,**D**). (**E**): The large tumour at laparotomy. (**F**): The surgical specimen. (**G**): The abdominal cavity after resection of the tumour. (**H**): Histopathologic examination established the diagnosis of a well-differentiated liposarcoma. Tumour slide with H/E stain and ×100 magnification ([25] with permission).

Adequate imaging is essential for appropriate treatment planning. The imaging method of choice for retroperitoneal masses is computed tomography (CT) of the abdomen and pelvis with intravenous contrast administration (Figure 1, Figure 2 and Figure 3). Alternatively, magnetic resonance imaging (MRI) can be used. Functional assessment of the contralateral kidney is important for treatment planning when en bloc resection of the ipsilateral kidney is considered and can be performed with contrast enhanced CT or a differential renal nuclear scan. Additional chest CT is sufficient for staging of the disease, since the lungs are the first sites to be involved in the case of DM [4,23,24].

The differential diagnosis of a retroperitoneal mass particularly includes, besides STS, lymphoma, metastatic adenocarcinoma, germ cell tumour, paraganglioma and benign soft tissue tumours. Hence, it is of critical importance to obtain a correct diagnosis before initiation of any treatment since the treatment of choice for these entities is completely different. Since imaging methods are rarely pathognomonic, diagnostic biopsy is necessary [4,23,24]. In case of germ cell tumours, serum tumour markers and examination of the testes and ovaries are helpful. Otherwise, only when the imaging is pathognomonic, as might be in the case of MRI of dedifferentiated or well-differentiated liposarcoma, and no preoperative treatment is considered, such a biopsy may be omitted [26]. Since differentiation among the various histological grades and subtypes might be of major importance in treatment planning, as will be discussed in detail below, a specialised sarcoma pathologist should examine the biopsy samples [4]. In addition to immunohistochemistry staining, cytogenetic analysis may be necessary to establish the correct diagnosis.

To solve the problem of the difficult accessibility of the mass, reduce the risk of damage to vital neurovascular structures, avoid biopsy of necrotic tissue and obtain tissue from the most suspicious and poorest differentiated component of the frequently heterogeneous mass, multiple image-guided percutaneous coaxial core needle biopsies (14 or 16 gauge) has been considered as the method of choice for obtaining tissue for histological and molecular examination [4]. Advanced MRI protocols with diffusion-weighted imaging models, T2 and T2* relaxometry, as well as spin coupling ratio may be helpful in the future to demonstrate the high-grade areas of heterogenous soft tissue tumours from which biopsy should be obtained [27,28,29]. Image-guided core needle biopsy, preferably via a retroperitoneal route, is safe and does not appear to increase the risk of LR or peritoneal recurrence [30,31,32,33,34]. Open and minimal invasive surgical biopsy should be avoided because of the risk of peritoneal seeding due to tumour rupture [4].

## 3. Treatment Plan

Various studies have shown that RPS management in expert, high-volume centres results in better outcome [33,35,36,37,38,39,40]. The treatment plan of each individual RPS patient should be discussed in a multidisciplinary sarcoma team, which should include a surgeon with experience in RPS resection, an expert sarcoma pathologist, a radiologist, a medical oncologist, a radiation oncologist and other surgical specialists depending on the extent of the tumour, often including urologists and vascular surgeons [4]. The dedicated imaging studies should be studied meticulously to determine the resectability of the tumour and to organise the appropriate surgical team. Although the only potentially curative treatment for primary RPS is surgery, the incidence of intra-abdominal recurrence remains high even in expert centres. The heterogeneity of RPSs with differences in biological behaviour, response to (neo)adjuvant treatment and oncological outcome according to grade and histological subtypes renders a standard treatment plan difficult. The treatment plan should be tailored in accordance with these parameters and the stage of the disease [4]. Moreover, the performance and nutritional status should be assessed and accordingly improved, since very large RPS may present late with symptoms of malnutrition, frailty and shortness of breath [4,41]. Although complete surgical resection remains the cornerstone of RPS treatment, the benefit of adjuvant and especially neoadjuvant treatment have been increasingly investigated to improve long-term disease control.

## 4. Surgical Approach

The best chance of cure is complete resection of the tumour en bloc with involved organs and structures and meanwhile minimizing the chance on microscopically tumour positive margins at the time of the initial operation. Several recent studies have demonstrated improved outcome when this often complex RPS surgery is performed in a high-volume expert centre [33,36,37,38,39]. Initial piecemeal and macroscopically incomplete resections at non-expert centres diminish the chance of disease control. Well-differentiated liposarcoma and the well-differentiated part of dedifferentiated liposarcoma may be easily overlooked at imaging and intraoperatively by a less experienced team, since it may be very difficult to differentiate a normal fatty tissue from a low-grade liposarcoma. Appreciation of asymmetry on preoperative imaging and complete resection of the retroperitoneal fatty tissue may be helpful in diminishing the risk of LR [4,5,14,15,16]. Additionally, when treated by an expert surgical team, tumour rupture may occur less often [5]. High-grade RPS may be friable, containing necrotic tissue, and hence should be handled with great care to avoid rupture and tumour spilling.

Generally, grossly incomplete resection of RPS is of no benefit and potentially harmful. It is only indicated in a palliative setting for selected highly symptomatic patients [4]. Unplanned grossly incomplete resection may be avoided in many cases with meticulous imaging review, careful planning and referral to an expert centre [4]. In a series of 322 RPS patients referred to a single expert centre, initial technical unresectability of the tumour was encountered in 6.2%, whereas patient-related parameters did not allow for major surgery in 5.6% [3]. Criteria for technical unresectability might include involvement of the superior mesenteric artery, aorta, coeliac trunk and/or portal vein, involvement of bone, growth into the spinal canal, invasive extension of retrohepatic inferior cava leiomyosarcoma into the right atrium and infiltration of multiple major organs such as liver, pancreas and/or major vessels [4]. Palliative surgery without tumour resection may be indicated in the case of gastrointestinal obstruction or bleeding.

In order to reduce the traditionally high recurrence rates, various strategies have been recently studied or are yet being studied. These concepts mainly include a more extended surgical approach, neoadjuvant radiotherapy (RT) and neoadjuvant systemic chemotherapy.

### Frontline Extended Surgery

**Figure 4:**
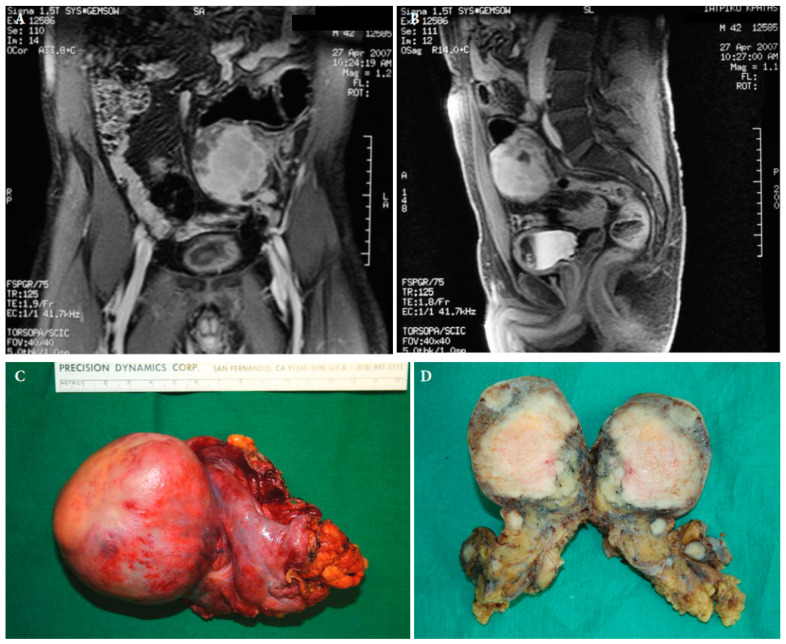
Magnetic resonance imaging of a retroperitoneal inflammatory myofibrosarcoma in a 42-year old male (**A**,**B**). Surgical specimen of the retroperitoneal tumour with satellite lesions (**C**,**D**) ([25] with permission).

Considering the high LR rate, a more extended surgical approach, which is especially considered for low-grade sarcomas, as outlined previously, was introduced approximately a decade ago [13,14,15,16,42]. A more aggressive approach in which the tumour en bloc with adherent structures is resected even if they are not overtly infiltrated is advocated. This concept is similar to that of resection of extremity STS with a rim of macroscopically normal tissue, aiming at better margins and improved LC. Preservation of specific organs and neurovascular structures should be considered on an individual basis which mandates weighing the potential of LC against the potential of acute and long-term morbidity when resected. In case of liposarcoma, the tumour cannot often be easily distinguished from normal retroperitoneal fat and ideally the entire ipsilateral retroperitoneal compartment, including all macroscopically normal fatty tissue, should be resected to obtain the adequate margins [13,14,15,16,42]. Moreover, RPS appears to be multifocal or presents satellite lesions (Figure 4) in approximately 20–30% of the cases and complete resection of the ipsilateral retroperitoneal fat may be helpful in reducing the risk of residual disease [43,44,45,46].

While in the past, cases of adjacency of the kidney only its capsule was removed at most, nowadays most experts support the removal of the kidney en bloc with the tumour mass. The case is similar for en bloc removal of adjacent bowel segments and spleen. Generally, with this approach, organs and structures whose removal en bloc with the tumour is associated with low morbidity, are liberally sacrificed when the distance to the macroscopic tumour is small. These organs and structures include kidneys, small bowel, large bowel, psoas muscle, spleen, tail of the pancreas and diaphragm. In contrast, organs and structures whose resection is associated with increased morbidity, such as the duodenum, head of the pancreas, vertebrae and major vessels, are only removed en bloc with the tumour when they are overtly infiltrated. The major vessels are exposed after removal of the adventitia. This more aggressive surgical strategy appears feasible with acceptable morbidity and promising oncologic outcome [13,14,15,16].

The teams of Silvie Bonvalot (Institute Gustave-Roussy and Institute Curie, Paris, France) and Alessandro Gronchi (Istituto Nazionale Tumori, Milan, Italy) have been pioneers of this approach. In a retrospective nationwide French study of 382 patients with RPS treated from 1985 to 2005 [13], the association of type of surgery and outcome was analysed. In a multivariate analysis, complete compartmental resection was associated with an approximately two-times lower risk of abdominal recurrence than after simple complete resection (*p* = 0.04) or en bloc resection with involved contiguous organs (*p* = 0.01). The risk of abdominal recurrence was almost twice as high after systemic re-excision, defined as the reoperation with systematic removal of the colon, kidney and psoas muscle en bloc after previous gross total resection, but this difference was not statistically significant (*p* = 0.18) due to the small number of patients in this treatment group. The 3-year abdominal recurrence rates for the above surgical approaches were 10%, 47%, 52% and 39%, respectively. In multivariate analysis, other factors associated with decreased abdominal recurrences included low grade (*p* = 0.008), no tumour rupture (*p* = 0.0001), negative histological margins (*p* = 0.008) and a high number of patients treated per centre (*p* = 0.04). Centres operating on higher number of patients performed compartmental resection more frequently (*p* < 0.0001) and had lower rates of intraoperative tumour rupture (*p* < 0.0001) than at low-volume centres. However, while in univariate analysis complete compartmental resection was associated with a four times better OS, type of surgery was not a statistically significant predictive factor for OS in multivariate analysis. In multivariate analysis, low grade, negative surgical margins, absence of tumour rupture and absence of gross residual disease were associated with increased overall survival. 

In another retrospective study [14], the results of surgery for 225 primary RPS and 106 first recurrences were analysed in the Istituto Nazionale Tumori for the periods before and after adoption of this more aggressive surgical strategy with liberal en bloc resections of adjacent organs and structures (136 patients in 1985–2001 and 152 patients in 2002–2007, respectively). The 5-year LR rate decreased from 48% in the early period to 29% in the more recent period of treatment (*p* = 0.0074). In multivariate analysis, the statistically significant determinants for decreased risk of LR were more recent period of treatment with a more aggressive surgical strategy (*p* = 0.0237), lower tumour grade (*p* = 0.0316) and histological subtype of liposarcoma (*p* = 0.0021). In an analysis of subgroups according to the tumour grade, a trend towards better LC for patients operated in the recent period with a more aggressive approach was observed for grade 1 and 2 (*p* =0.0636 and *p* = 0.0656, respectively), whereas for grade 3 tumours no benefit was observed (*p* = 0.272). Moreover, in analysis according to histological type, only patients with liposarcoma had a significantly better 5-year LC with extended surgery (64% vs. 39%, *p* = 0.0070). In patients with leiomyosarcoma, both surgical approaches were associated with similar LR risk. Regarding the risk of DM, remarkably, a higher 5-year DM rate was found in the recent period of treatment (22% vs. 13%, *p* = 0.0125). This increased incidence of DM was only observed for the high-grade sarcomas. In an analysis of subgroups according to grade, no difference in terms of occurrence of metastatic disease was observed for grade 1 and 2 tumours between the treatment periods, whereas a highly significant increase in the number of patients with metastases for grade 3 tumours was noted in the recent period (*p* = 0.0002). The explanation for this observation remains unclear. Factors such as immune depression after major surgery and the release of growth factors locally might be possible factors. However, the authors note that those who developed metastases were not over-represented among the fraction of those who underwent extended surgery, andhence, they attribute this trend to a prognostic shift in the patient population. As expected, low histological grade and histological subtype (liposarcoma vs. leiomyosarcoma) were highly associated with increased distant-disease-free survival in a multivariate analysis. Although the 5-year OS was higher in the recent period (60% vs. 51%), the difference was not statistically significant. In general, the median follow-up period was too short in the recently treated group of patients (32 months) in order to demonstrate difference in long-term survival. With the aim of demonstrating OS benefit, the same centre reported data after longer follow-up in a later publication [15]. Frontline extended surgery (191 patients, 48 months median follow-up) was associated with 5 years OS of 67% and a 5-year LR-free survival of 72%, while after traditional surgery (140 patients, 127 months median follow-up) these rates were 48% (*p* = 0.009) and 51% (*p* = 0.001), respectively. The 5-year risk of DM remained higher in the recently treated patients (25% vs. 12%, *p* = 0.005). Again, DM were mainly observed in high-grade tumours and histological types other than liposarcoma. The above retrospective data support the opinion that aggressive surgery with liberal en bloc resections of adjacent organs and structures may not be beneficial for high-grade tumours due to their tendency to give rise to DM and subsequent death. In contrast, extended surgery seems to be valuable especially in patients with a grade 1 or 2 tumour and/or liposarcoma.

In a most recent retrospective study of 109 patients who underwent frontline extended, multivisceral surgery for retroperitoneal liposarcoma [47], clinically relevant histological organ involvement was in 78% and 84% of patients with well-differentiated and dedifferentiated liposarcoma, respectively. Those high rates support the concept of frontline extended, multivisceral surgery in retroperitoneal liposarcoma.

To demonstrate the safety of this novel surgical approach, both centres that initiated this approach (Institute Gustave-Roussy, Paris, France and Istituto Nazionale Tumori, Milan, Italy) gathered their experience and analysed the collected data [16]. A total of 249 consecutive patients with primary RPS had been treated with a frontline extended surgical approach. Complete macroscopic resection of the tumour, with a median size of 7 cm, was 93%. The median number of resected organs en bloc with the tumour was two. The operative mortality was 3%, while postoperative morbidity requiring an invasive therapeutic procedure was noted in 18% of the patients and surgical re-intervention was necessary in 12% of the patients. Major morbidity included anastomotic leakage (9%), intra-abdominal abscess (4%), postoperative bleeding (2%), wound dehiscence (2%), pulmonary embolism (0.4%) and lower limb compartmental syndrome (0.4%). No patient received a permanent stoma, and no patient developed renal failure or femoral neuropathy. An almost three-fold increased risk (*p* = 0.007) of morbidity was observed when more than three organs were resected concomitantly. After adjustment for the number of organs resected, only resection of large vessels, the stomach and small bowel (i.e., duodenum) remained to be associated with increased morbidity at univariate analysis. The most common removal of colon, kidney and psoas muscle was not associated with increased morbidity. Similar postoperative morbidity was noted in other studies. The combined experience from expert centres of the Transatlantic Retroperitoneal Sarcoma Working Group (TARPSWG), including 1007 consecutive operations, revealed severe postoperative complications in 16% of the patients and a 30-day postoperative mortality rate of 1.8% [48]. In a most recent retrospective study of a French referral centre [49], including 265 extended, multivisceral resections for primary or recurrent RPS, severe postoperative adverse events were reported in 19% of the patients, 6% underwent an iterative laparotomy and 2% died within 90 days. Blood transfusion requirement, operation time, number of digestive anastomoses, and pancreas and/or major arterial resection were associated with higher surgical morbidity. Preoperatively known parameters that were significant predictors of major complications in multivariate analysis included male gender, performance status, dedifferentiated liposarcoma histology and low serum albumin level. Preoperative treatment was not associated with increased severe morbidity. The appreciation of preoperatively known parameters that impact safety could moderate the extent of surgery, specifically the resection of adherent organs that are not overtly invaded. In all studies [16,48,49], postoperative morbidity did not seem to have an adverse impact on oncological outcome, neither on LR nor on distant recurrence and OS. 

Chronic morbidity after primary extended resection for RPS may be seen after resection of the psoas adjacent to the femoral nerve and nephrectomy [50].The majority of the patients with injury to the femoral nerve exhibit some sensory impairment of the limbs, but severe chronic pain and lower limb functional impairment are rare. Additionally, it appears that long-term renal function is not significantly impaired when an ipsilateral nephrectomy is performed. Although a formal comparison with postoperative morbidity of a more conservative approach of primary RPS has never been systematically analysed, postoperative morbidity seems similar for both approaches [4].

Resection of major vessels is occasionally needed in surgery for RPS. Although vascular resection en bloc with the tumour is associated with increased morbidity, vascular resection to facilitate adequate resection of RPS has an acceptable long-term patency rate of the vascular reconstruction [51,52,53,54,55]. Although the encasement of the vascular bundle does not represent a contraindication to surgery, there is an association with a high metastatic risk by virtue of the locally advanced nature of the disease and this should be considered when planning treatment. 

In conclusion, it seems that superior LC is achieved by extended, multivisceral surgery in low-grade sarcoma and liposarcoma. For leiomyosarcoma, there seems to be no clear benefit from a more aggressive surgical approach, most probably because tumour borders are more clearly defined and systemic disease is frequently encountered [4]. Solitary fibrous tumour generally exhibits a low risk of LR and extended surgery seems unnecessary in most cases [18,19]. Severe morbidity and mortality after this surgical approach appears to be relatively low and acceptable. In general, the morbidity and mortality of this extended surgical strategy seems comparable to that of major abdominal surgery.

## 5. Perioperative Radiotherapy

In STS of the extremities, two randomised trials have demonstrated that adjuvant RT significantly decreases LR risk [56,57]. Extrapolation of the results of these studies has led to the application of adjuvant RT in the treatment of RPS. Owing to its rarity and the long follow-up required due to its commonly indolent biological behaviour, it has been difficult to assess the efficacy of adjuvant RT for primary RPS. Some non-randomised comparative studies have shown a decreased risk of LR with adjuvant RT in RPS. A retrospective study of the French Cancer Federation Sarcoma Group [7] demonstrated an increase of 5-year LC from 23% to 55% (*p* = 0.0021) by adding RT to surgery. In another study [58], the prospective database of a referral centre which did not use adjuvant RT (Memorial Sloan Kettering Cancer Center, New York, NY, USA) was compared with that of one who did routinely use adjuvant RT after resection of a retroperitoneal STS (Massachusetts General Hospital, Boston, MA, USA). The 32 patients who received adjuvant RT exhibited a significantly higher 5-year LR-free survival rate than the 172 patients who only underwent surgery (91% vs. 65%, *p* = 0.03 in multivariate analysis). However, the disease-specific survival was not significantly different (93% vs. 85%, *p* = 0.3), and the perioperative morbidity was significantly higher among the patients who also received RT (44% vs. 16%, *p* = 0.004). In a French study, postoperative RT seemed to further benefit local outcome even after extended surgery for RPS [59]. In the Italian comparative study of standard versus more aggressive surgery [15], the addition of RT, administered preoperatively or postoperatively, seemed to result in LR reduction (*p* = 0.023) and a strong trend towards increased OS (*p* = 0.050). In large case–control, propensity score-matched analyses of the US National Cancer Database (2003–2011), including 9068 patients with primary RPS, among whom 563 had received preoperative, 2215 postoperative and 6290 no RT, there was demonstrated to have improved OS for preoperative RT vs. no RT (median 110 months vs. 66 months, HR = 0.70, *p* < 0.0001) and for postoperative RT vs. no RT (89 vs. 64 months, HR = 0.78, *p* < 0.0001) [60]. Also, in a recent meta-analysis [61], including 10 studies and a total of 5237 RPS patients who received surgery alone and 3568 surgery and pre- or postoperative RT, 5-year LR-free survival and 5-year OS were significantly improved with the addition of RT (HR = 0.33, *p* < 0.00001 and HR = 0.69, *p* < 0.00001). While pre- and postoperative RT had a similar beneficial effect on OS (for both HR = 0.69), preoperative RT seemed to have higher impact on LR reduction than postoperative RT (HR = 0.19 and HR = 0.49, respectively).

A major problem with adjuvant RT for RPS is its toxicity to the bowel and to a lesser extent to the kidney(s), the liver, the ureter(s), urinary bladder, spinal cord and major peripheral nerves. The toxicity may be decreased with intraoperative RT (IORT), brachytherapy, placement of tissue expanders to keep the bowel out of the radiation field, preoperative RT and modern RT techniques such as intensity modulated radiation therapy (IMRT) with less radiation emitted to structures outside the target field [62,63]. IORT has no proven benefit. It may be used for margins considered at risk, but the field is usually too large for practical application, and the dose that can be delivered too small to omit postoperative external beam RT [62]. In a small randomised trial [64], disabling radiation-related enteritis was significantly less frequent after 20 Gy intraoperative and 35–40 Gy postoperative external beam RT when compared with 50–55 Gy postoperative external beam RT (13% of 15 patients vs. 50% of 20 patients, *p* < 0.05). However, radiation-related peripheral neuropathy was much more frequent among those who received IORT (60% vs. 5%, *p* < 0.001). After more than 5 years of follow-up, the LR rate was lower in the IORT group of patients (40% vs. 80%, *p* < 0.001), and the median survival was similar for both groups of patients. The substantial toxicity and the high LR rate in comparison with more recent series with modern RT techniques have impeded its use in RPS. An alternative approach to reduce toxicity is the administration of RT before instead of after surgery [62,63]. Due to intact blood supply and microcirculation before surgery, a lower radiation dose is sufficient, while a smaller radiation target field is adequate in the absence of wide surgical dissection planes. Lower dose and smaller field reduce the radiation exposure to adjacent healthy organs and structures. Moreover, less small bowel toxicity is exhibited due to the tumour itself keeping the bowel outside the radiation target field and due to the lack of adhesions that may cause a bowel segment to be exposed to radiation during the entire RT course. Additional potential advantages of preoperative RT, when compared with postoperative RT, include decreased risk of intraoperative spread of viable malignant cells and tumour size reduction. The potential disadvantages are the delay of surgery, difficulty in histological examination in the case of major or complete response and increased wound complications. In extremity STS, a randomised trial [65,66,67] comparing preoperative with postoperative RT has demonstrated similar LC with decreased long-term morbidity (i.e., oedema, fibrosis and functional impairment), increased wound complication rate and a small unexplainable increase in OS.

Considering the retrospective data of previous studies, whose evidence has been limited by using RT usually for tumours that are smaller in size, located at more favourable sites, easier to irradiate and resect as well as treated in single academic centres, and less expected toxicity of preoperative rather than postoperative RT, randomised trials were designed to determine the definite benefit of preoperative 3D conformal RT (3DCRT) or IMRT in patients with primary RPS. The ACOSOG-Z9031 study (NCT00091351) and the MSKCC study (NCT00131898) prematurely closed due to poor accrual. The EORTC performed the multi-centre randomised STRASS trial (NCT01344018) [68]. A total of 31 centres in 13 countries in Europe and North America participated. Although266 patients were initially enrolled, eventually 128 patients underwent multivisceral en bloc curative-intent surgery alone and 119 received both 50.4 Gy preoperative external beam RT and this type of surgery. Serious adverse events were more common in the group of patients who received the combined treatment (24% vs. 10%). The most common grade 3–4 adverse events included lymphopenia (77% vs. 1%), anaemia (12% vs. 8%) and hypoalbuninaemia (12% vs. 4%). One patient died due to a treatment-related adverse event being a RT-induced gastropleural fistula. Although 11% of the patients exhibited local disease progression during preoperative RT, the vast majority still underwent macroscopically complete resection. After a median follow-up of 43.1 months, the median abdominal recurrence-free survival was not different among the treatment arms (5.0 vs. 4.5 years, respectively, *p* = 0.95). OS did not differ between the groups of patients either. However, one should consider that the follow-up period is yet short for this disease with a frequent indolent natural behaviour. The investigators concluded that preoperative RT should not be considered as standard of care treatment for all primary RPS. When the compliance to the RT protocol was considered, the 3-year abdominal recurrence-free survival was significantly higher when RT was administered according to this protocol than when deviations, mainly incorrect volume delineations, were observed (66.8% vs. 49.8%, HR = 2.32, *p* = 0.008) [69]. Hence, high quality of preoperative RT seems to contribute to improved LC. 

However, in the heterogenous group of RPSs, the efficacy of (neo)adjuvant RT may vary among histological subtypes. Some studies have focused on the impact of RT in liposarcoma, the most common histological type of RPS. The TARPSWG retrospectively analysed the efficacy of the addition of RT to surgery for primary localised retroperitoneal liposarcoma in 8 high-volume centres [70]. Among the total of 607 patients, 234 had well differentiated liposarcoma, 242 grade 1 or 2 dedifferentiated liposarcoma and 131 grade 3 dedifferentiated liposarcoma. RT was delivered, mainly preoperatively, in 19.7%, 34.7% and 35.1%, respectively. In univariate unadjusted analysis, the 8-year LR rates were significantly lower when RT was added to surgery (11.8% vs. 39.2%, *p* = 0.011; 29% vs. 56.7%, *p* = 0.008; 29.8 vs. 43.7%, *p* = 0.025, respectively). However, this significant impact of RT on LC was lost after accounting for imbalances in prognostic variables using propensity scores. RT had also no clear effect on the incidence of DM and on overall survival in this study. Most recently, in a propensity-matched analysis of 3911 patients with primary localised retroperitoneal liposarcoma in the US National Cancer Database (2004–2017), the impact of RT was studied merely on OS and not on LR or disease-free survival [71]. A total of 2252 patients had well-differentiated liposarcoma and 1659 dedifferentiated liposarcoma, without distinction of grading. After a median follow-up of 4.1 years, median OS was 10.7 years. After propensity score matching, the addition of RT, either postoperatively or preoperatively, did not significantly improve OS in either liposarcoma group. However, in meta-analysis, there was a trend for better OS with preoperative RT in the well-differentiated liposarcoma group (HR = 1.80, 95% CI 0.95–3.42, *p* = 0.067). Notably, the follow-up period was short for a disease in which the natural history occurs over many years. In this context, an endpoint as disease recurrence might be more useful for assessing the impact of a local adjuvant treatment. In an ad hoc analysis of patients with liposarcoma in the STRASS trial, preoperative RT was associated with a trend towards improvement in the 3-year abdominal recurrence-free survival (71.6% vs. 60.4%, HR = 0.64, 95% CI 0.40–1.01) [68]. This trend was not seen for leiomyosarcoma and other subtypes. Nevertheless, the individual subgroups were small and such sub-analyses should be regarded with caution. To increase the number of patients for analysis, 202 patients from the STREXIT observational study who were treated for primary RPS with surgery with or without preoperative RT during the STRASS recruiting period in the same centres, but not enrolled in the STRASS study for various reasons, were added after propensity score matching to the cohort of the STRASS randomised trial [72]. In the pooled cohort analysis, preoperative RT was associated with improved 5-year abdominal recurrence-free survival in patients with well-differentiated liposarcoma and grade 1 or 2 dedifferentiated liposarcoma (n = 266, 65.8% vs. 56.0%, HR = 0.63, 95% CI 0.40–0.97), but not in patients with grade 3 dedifferentiated liposarcoma or leiomyosarcoma. Similarly, in this analysis, no impact on OS or DM-free survival was observedafter a relatively short median follow-up of approximately 40 months.

In conclusion, patients with well-differentiated liposarcoma and grade 1 or 2 dedifferentiated retroperitoneal liposarcoma seem to potentially benefit from preoperative RT with respect to decrease in abdominal recurrence risk. Since local liposarcoma recurrences can usually be re-operated and these tumours are prone to late recurrences, longer follow-up of the above series is needed to determine any definite effect on OS. Preoperative RT does not seem to be indicated in high-grade RPS and leiomyosarcoma. Other histological RPS subtypes, such as malignant peripheral nerve sheath tumour (MPNST) and solitary fibrous tumour, were underrepresented in the above studies and data available on the benefit of preoperative RT are very limited. Retrospective studies suggest that perioperative RT in patients with primary extra-meningeal solitary fibrous tumour may reduce the risk of LR, especially in the case of involved surgical margins and a high mitotic count, although there seems to be no impact of RT on overall survival [73,74].

Various attempts are ongoing to improve the response to preoperative RT. The randomised Act.In.Sarc trial evaluated the safety and efficacy of the intratumoural administration of a radioenhancer, hafnium oxide nanoparticle NBTXR3, during preoperative RT in locally advanced sarcoma of the extremities and trunk wall [75,76]. The administration of NBTXR3 doubled the complete response rate (16% vs. 8%, *p* = 0.044) [75], without having a negative impact on safety or health-related quality of life [76]. Unfortunately, the accessibility for intratumoural administration and tumour size hinder its application in RPS. Limited RT efficacy may be caused by tumour microenvironement hypoxia, which is definitely anticipated in large RPS and results in decreased DNA damage and by acquired immune tolerance due to amplified PD-L1 expression [77]. Due to the hypoxic tumour microenvironment and amplified PD-L1 expression, the response to RT is impaired. Recent promising experimental studies have focused on targeting the hypoxic tumour environment as well as improved anti-PD-L1 efficacy with specifically developed nanoparticles to improve the efficacy of (immune-RT [77,78]. Other techniques to improve the efficacy of preoperative RT are being investigated in phase I/II trials, such as dose intensification only to the posterior wall for high-risk margins of RPS through “dose painting” with proton or photon IMRT (NCT01659203) [79]. Interest in concurrent chemo-RT, particularly in the subgroup of primary RPS with high metastatic potential and risk of distant disease progression during preoperative RT, has led to inclusion of patients with retroperitoneal dedifferentiated liposarcoma and leiomyosarcoma in the ongoing multicentre phase I/II TRASTS trial (NCT02275286) to test the efficacy of trabectidin together with low-dose RT [79].

## 6. (Neo)Adjuvant Chemotherapy

Even with optimal LC, distant metastatic failure remains a significant issue in high-grade RPS and certain histological subtypes, such as leiomyosarcoma. Hence, effective adjuvant systemic treatment options are needed to improve survival for those RPS patients. In general, adjuvant systemic chemotherapy appears of limited benefit in primary (high-risk) STS [80,81]. No randomised study has yet been published including only RPS. In a meta-analysis of 18 randomised trials [80], which included STS at any site, adjuvant systemic chemotherapy significantly reduced the risk of LR and overall recurrence (*p* = 0.02 and *p* = 0.0001, respectively), but not of OS (*p* = 0.09). Only patients who received the combination of doxorubicin and ifosfamide displayed a statistically significant benefit in OS (*p* = 0.01). However, the 6% absolute increase in survival was achieved at the cost of considerable toxicity. In a more recent multicentre randomised EORTC trial [81], 301 patients with intermediate and high-grade STS at any location underwent surgery alone or followed by systemic chemotherapy with doxorubicin and ifosfamide. No significant differences in locoregional disease control, disease-free survival and overall survival were noted. However, when the results of this randomised trial were revisited with assessing the risk on systemic recurrence using a validated prognostic nomogram tool called Sarculator [82,83], adjuvant chemotherapy significantly reduced risk of recurrence and death in the group of patients who had a predicted probability of 10-year OS less than 60% the (HR = 0.49, 95% CI 0.28–0.85 and HR = 0.50, 95% CI 0.30–0.90, respectively) [84]. In the groups with a higher predicted probability of OS, no improvement in outcome was observed with adjuvant chemotherapy. Hence, adequate selection of high-risk patients for adjuvant systemic treatment seems to be of utmost importance. This may help to reconcile the disparate results of clinical studies on adjuvant/neoadjuvant chemotherapy in STS.

Preoperative or neoadjuvant systemic chemotherapy has the potential advantages of avoidance of delay in starting systemic chemotherapy due to surgical complications, avoidance of diminished chemotherapy tolerability after major surgery, early treatment of micrometastases, reduction in tumour size resulting in organ preservation during surgery, testing the tumour’s biology that can prevent aggressive, futile surgery in those who progress during treatment, and in vivo assessment of treatment response [85]. Furthermore, preoperative treatment may be relevant in order to downsize technically unresectable or borderline resectable RPS and render it more amenable to safe and grossly complete resection. In a former randomised EORTC trial [86] on the benefit of preoperative systemic chemotherapy for high-risk STS, RPS patients were not included. The 5-year OS and disease-free survival were similar after surgery with or without preoperative systemic chemotherapy with doxorubicin and ifosfamide. Unfortunately, LR risk was not separately analysed.

Based on the positive results of its randomised trial on the adjuvant treatment with 5 cycles of high-dose epirubicin and ifosfamide for high-risk STS of the extremities and the girdles, in which, however, the dose-intensity of the last two cycles was suboptimal [87], the Italian in collaboration with Spanish Sarcoma Groups conducted a randomised trial comparing three preoperative with three preoperative and two postoperative cycles of this drug regimen [88]. Three cycles of full-dose preoperative chemotherapy were not inferior to five cycles (three preoperative and two postoperative) in terms of OS, incidence of DM and LR risk.

Acknowledging the fact that not every histological subtype responds similarly to the same (standard) chemotherapeutics, a multicentre randomised trial investigated 3 cycles of histotype-tailored versus 3 cycles standard systemic neoadjuvant chemotherapy (epirubicin and ifosfamide) in 287 patients with high-risk STSs of the extremities and the trunk wall [89]. Histotype-tailored chemotherapy consisted of trabectedin for myxoid liposarcoma, gemcitabine plus dacarbazine for leiomyosarcoma, ifosfamide plus etoposide for malignant peripheral nerve sheath tumour, high-dose ifosfamide for synovial sarcoma, and gemcitabine plus docetaxel for undifferentiated pleomorphic sarcoma. Surprisingly, the histotype-tailored approach was inferior to the standard chemotherapy regimen in terms of disease-free survival and OS after a median follow-up of 12 month, and the study was prematurely closed [89]. After a longer median follow-up period of 52 months, OS remained inferior for the histotype-driven chemotherapy, but disease-free survival became similar [90]. Most recently, a post hoc analysis of the patients in the above trial investigated the efficacy of standard neoadjuvant chemotherapy according to prognostic stratification based on the Sarculator nomogram for STS [91]. In the high-risk group with a predicted 10-year overall survival of less than 60%, the observed overall survival was significantly better than the predicted (*p* = 0.04). Improvement in predicted overall survival with neoadjuvant therapy was not observed for histotype-tailored chemotherapy and low-risk STSs (predicted 10-year overall survival of ≥60%). Hence, it seems that neoadjuvant chemotherapy with a combination of anthracycline (epirubicin or doxorubicin) with ifosfamide is a valid treatment option for high-risk STS localised on the extremities or the trunk wall.

However, these results cannot be extrapolated to RPS since the predominant histology types are different, and even minor progression may render resectable tumours irresectable conversely to extremity sarcomas, and chemotherapy-related adverse events in the often frail and malnourished RPS patient may result in a contraindication for major surgery [5]. In two analyses of the US National Cancer Database (1998–2011 [92] and 2004–2013 [93]), only 10–18% of the curatively resected RPS patients received adjuvant chemotherapy, of which 11% in the neoadjuvant setting. In propensity score-matched cohort analyses, adjuvant chemotherapy was not associated with improved survival, not even in high-risk patients. There was a trend towards improved overall survival in spindle cell, giant cell and synovial sarcoma, but these histological subtypes are rare in RPS. In a recent multi-centre retrospective TARPSWG study [94], the role of neoadjuvant chemotherapy in 158 high-risk RPS patients who underwent subsequently curative surgery was evaluated. A median of 3 cycles of neoadjuvant chemotherapy had been administered. The regimen varied, but most contained anthracycline. Regarding the radiological response, 23% of patients exhibited a partial response, 56% stable disease and 21% progressive disease, while no complete response was noted. While the patients with progressive disease in this study were still operated, the resectability after progressive disease should be considered with caution since the patients who initially received neoadjuvant chemotherapy, but were not eventually operated, were not evaluated in this study. As expected, patients with progressive disease during neoadjuvant chemotherapy had the worse overall survival. No differences of LR and DM rates were observed according to the type of radiological response. While the kind of regimen, anthracycline plus ifosfamide or another, had no impact on radiological response in grade 3 dedifferentiated liposarcoma, anthracycline plus dacarbazine was associated with better outcome in leiomyosarcoma.

Only a prospective randomised trial will be able to establish the definite role of neoadjuvant systemic chemotherapy in high-risk resectable RPS. In the ongoing STRASS 2 trial (NCT 04031677) [79], RPS patients with grade 3 dedifferentiated liposarcoma and leiomyosarcoma, the histological RPS subtypes with a high risk of distant metastases, are randomised to neoadjuvant systemic chemotherapy and surgery versus surgery alone. Since considered adequate from previous prospective studies on extremity and trunk STS and in concordance with the median number in the retrospective TARPSWG study in RPS, only 3 cycles of systemic chemotherapy are administered preoperatively. Based on the data of the latter retrospective multicentre study, patients with grade 3 dedifferentiated liposarcoma receive doxorubicin plus ifosfamide and those with leiomyosarcoma receive doxorubicin plus dacarbazine. The primary endpoint is disease-free survival, whereas enrolment of 250 patients is required and estimated to be completed in 2029.

An interesting randomised trial on the benefit of regional hyperthermia in perioperative systemic chemotherapy with etoposide, doxorubicin and ifosfamide for high-risk STS at any site, including RPS, demonstrated improved response of the tumour to neoadjuvant therapy with the addition of hyperthermia (*p* = 0.02) [95]. After a median follow-up of 11.3 months, the addition of regional hyperthermia to systemic chemotherapy appeared to improve local progression-free survival (HR = 0.65, 95% CI 0.49–0.86, *p* = 0.002) and overall survival (HR = 0.73, 95% CI 0.54–0.98%, *p* = 0.04) [96]. A subgroup analysis of patients with completely resected high-risk abdominal and retroperitoneal sarcoma demonstrated that the 76 patients undergoing systemic chemotherapy with regional hyperthermia experienced lower LR and overall recurrence rates than the 73 patients who only underwent systemic chemotherapy (5-year LR-free survival 56% vs. 45%, *p* = 0.044; 5-year disease-free survival 34% vs. 27%, *p* = 0.040) [97]. There was no significant difference in overall survival between the groups of patients after 5 years. For high-risk RPS patients who are candidates for neoadjuvant treatment, the addition of regional hyperthermia may be an interesting treatment option. However, a serious limitation for its application is the fact that the technology to perform regional hyperthermia is not widely available.

Regarding the role of immunotherapy in the treatment of RPS, currently response rates to immune checkpoint inhibitors have been very poor [98]. In the Alliance A091401 trial [99], objective response was observed in 5% and 16% of RPS patients with nivolumab monotherapy and the combination of nivolumab and ipilimumab, respectively. This might be explained by the rare incidence of mismatch repair (MMR) deficiency and the low tumour mutational burden in STSs [98]. The heterogeneity of STS carries significant implications as sarcoma subtypes vary in mutational burden, immune infiltrate, and receptor status, all of which directly affect response to immunotherapy. None of the histological subtypes commonly observed in the retroperitoneum are associated with a high tumour mutational burden, and the commonly found well differentiated liposarcoma exhibits even the lowest tumour mutational burden of all subtypes [98]. A French Phase 2 trial (TORNADO, NCT04968106) plans to examine the effect of neoadjuvant chemotherapy with retifanlimab, a humanised PD-L1 inhibitor, on histologic response for previously untreated resectable RPS of any histology [79]. Further studies are definitely needed to harness and manipulate the tumour microenvironment and immune composition of these tumours in order to successfully apply immunotherapy [98]. In the future, other strategies may be more effective, such as tumour selective metabolic reprogramming, via mitochondria oxidative phosphorylation depression, which down-regulates PD-L1 and reactivates immunotherapy, opening a potential window for mitochondrial immunotherapy [100].

In search for more effective new systemic treatments for these rare tumours, there is a need for classification beyond histological features including molecular classifications to predict the natural history and responsiveness to treatments. Further research is warranted to identify reliable predictive biomarkers to refine patient selection for chemotherapy, targeted treatment and immunotherapy.

## 7. Treatment of Local Recurrence

Surgery remains the treatment of choice for LR of RPS in the absence of systemic disease. Generally, the same principles as for surgery of primary RPS are advocated. First LR is resectable in approximately 55% of the cases and associated with a 6-year overall survival of 54% [101,102]. The feasibility of surgery decreases with each further recurrence. The most common histological subtype operated on first LR is dedifferentiated liposarcoma [101,102]. In specialised centres, morbidity and mortality are similar to that of surgery for primary RPS, with a 16% incidence of severe complications and a 90-day postoperative mortality of 0.4% [102]. Similar to the observation of surgery for primary RPS, major morbidity does not seem to be associated with impaired oncological outcome [102].

The decision to proceed to curative resection of first local RPS recurrence must be taken after consideration of the nature of the previous operation, expected completeness of the new resection, histological subtype and grade, disease biology as well as patient’s performance status and comorbidities [103]. In fast-growing LR, the benefit of surgery may be minimal or nil. A retrospective study demonstrated that patients with LR that grew less than 0.9 cm per month benefit from surgery, while those with faster growing recurrences do not [104].For patients with a subsequent recurrence, absence of systemic disease and disease-free interval after surgery for the previous recurrence is more important rather than histology or grade [103].Treatment delay is appropriate for patients who are asymptomatic or have minimal symptoms, in cases wherein the tumour burden is limited and does not pose a major threat of complications with progression, and in cases wherein tumour growth would not substantially change the scope of subsequent resection [103].

The decision on how to treat local RPS recurrence is complex, individualised to the patient and should be taken after discussion by a multidisciplinary team. Outcomes for patients with recurrent RPS remain poor and data on the benefit of (neo)adjuvant treatments are lacking, but extrapolation from results of the aforementioned studies of primary RPS may be helpful. For a more in-depth analysis of the optimal approach on recurrent RPS, we would like to refer to a most recently updated consensus publication [103].

## 8. Conclusions and Future Directions

The management of primary RPS may be improved with adequate preoperative imaging and histological diagnosis, case discussion in a multidisciplinary team and treatment in a referral centre. While surgical resection remains the mainstay treatment for primary localised RPS, outcome is worse than for STSs at other sites. LR after surgery is more frequent mainly due to anatomical constraints, its large size, indistinct tumour borders and thinness of the overlying peritoneum that make wide local resection, as performed in extremity and trunk wall STS, usually unfeasible. In contrast to most malignant diseases, LR is the leading cause of death for these usually low-grade tumours. In high-grade tumours, the risk of metastatic disease is a major concern.

Due to its heterogeneity, there is not a one-size-fits-all treatment for primary RPS, and emerging therapeutic strategies aim to provide optimal treatment for various histological types and malignancy grades (Box 1). There is sufficient evidence that frontline extended surgery with compartmental resection including all ipsilateral retroperitoneal fat and liberal en bloc resection of adjacent organs and structures, even if they are not macroscopically involved, increases LC in low-grade sarcoma and liposarcoma, but not in leiomyosarcoma for which complete macroscopic resection seems sufficient. Additionally, preoperative RT is not indicated for all RPSs, but seems to be beneficial in well-differentiated liposarcoma and grade I/II dedifferentiated liposarcoma and probably in solitary fibrous tumour. Various attempts are ongoing to improve the response to preoperative RT, such as chemo-RT, immune-RT and novel RT techniques. Whether neoadjuvant chemotherapy is of benefit in high-grade RPS is subject of the ongoing randomised STRASS 2 trial, and the results are eagerly awaited. Currently, response rates to immune checkpoint inhibitors have been very poor. Studies are ongoing to improve the efficacy of immunotherapy. Better patient selection, quality of surgery and perioperative treatment resulted in increased overall survival and reduced postoperative mortality for patients undergoing resection for primary RPS in 10 referral centres over a recent 15-year period [105]. Since randomised trials are difficult to perform for these rare and heterogenous RPSs despite impressive global collaborations, data of a large prospective multicentre observational study like the REtroperitoneal SArcoma Registry (RESAR) may be helpful in the future in identifying the optimal treatment for patients with primary RPS [106]. Personalised, histology-tailored multimodality treatment is promising and will likely further evolve as our understanding of the molecular and genetic characteristics within histologic types improves.

Box 1 **Summary of the currently major emerging issues in RPS treatment.**

- The traditional approach with surgery only for primary localised RPS is associated with a high local recurrence rate for low-grade tumours and a high incidence of distant recurrences for high-grade tumours and multimodality approach may improve oncological outcome.
- Current treatment of primary localised RPS is mainly defined by probability of complete resection (R0), histological subtype and malignancy grade.
- Hence, preoperative histological diagnosis and subsequent discussion in a multidisciplinary team is essential for an optimal treatment plan.
- Frontline extended surgery with compartmental resection and multivisceral surgery (i.e., liberal en bloc resection of adjacent organs and structures) increases local tumour control in low-grade sarcoma and liposarcoma and is associated with acceptable morbidity in expert centres.
- Preoperative radiotherapy seems to be beneficial in well-differentiated liposarcoma and grade I/II dedifferentiated liposarcoma, and probably in solitary fibrous tumour.
- Various attempts are ongoing to improve the response to preoperative RT, such as chemo-RT, immune-RT and novel RT techniques.
- Whether neoadjuvant chemotherapy is of benefit in high-grade RPS remains unclear from retrospective data and is subject of the ongoing randomised STRASS 2 trial.
- Currently, response rates to immune checkpoint inhibitors have been very poor and further studies are definitely needed to harness and manipulate the tumour microenvironment and immune composition of these tumours in order to successfully apply immunotherapy.
- Personalised, histology-tailored multimodality treatment is promising and will likely further evolve as our understanding of the molecular and genetic characteristics within RPS improves.

## Data Availability

The data presented in this study are available in this article.
